# Metabolite-mediated interactions and direct contact between *Fusobacterium varium* and *Faecalibacterium prausnitzii*

**DOI:** 10.1186/s40168-025-02168-w

**Published:** 2025-07-28

**Authors:** Koji Hosomi, Satoko Maruyama, Tsubasa Matsuoka, Mari Furuta, Yoko Tojima, Keita Uchiyama, Makiko Morita, Hitoshi Kawashima, Toshiki Kobayashi, Jun Kunisawa

**Affiliations:** 1 Graduate School of Veterinary Science, Osaka Metropolitan University, 1-58 Rinku-Oraikita, Izumisano, Osaka 598-8531 Japan; 2 Laboratory of Vaccine Materials, Microbial Research Center for Health and Medicine, National Institutes of Biomedical Innovation, Health and Nutrition, 7-6-8 Saito-Asagi, Ibaraki, Osaka 567-0085 Japan; 3 Research and Development Department, Hakubaku Co., Ltd., 4629 Nishihanawa, Chuo, Yamanashi 409-3843 Japan; 4 Laboratory of Gut Environmental System, Microbial Research Center for Health and Medicine, National Institutes of Biomedical Innovation, Health and Nutrition, 7-6-8 Saito-Asagi, Ibaraki, Osaka 567-0085 Japan; 5 Graduate School of Medicine, Osaka University, 2-2 Yamadaoka, Suita, Osaka 565-0871 Japan; 6 Graduate School of Pharmaceutical Sciences, Osaka University, 1-6 Yamadaoka, Suita, Osaka 565-0871 Japan; 7 Graduate School of Dentistry, Osaka University, 1-8 Yamadaoka, Suita, Osaka 565-0871 Japan; 8 Graduate School of Science, Osaka University, 1-1 Machikaneyamacho, Toyonaka, Osaka 560-0043 Japan; 9 International Vaccine Design Center, The Institute of Medical Science, The University of Tokyo, 4-6-1 Shirokanedai, Minato-Ku, Tokyo 108-8639 Japan; 10 Department of Microbiology and Immunology, Kobe University Graduate School of Medicine, 7-5-1 Kusunoki-Cho, Chuo-Ku, Kobe, Hyogo 650-0017 Japan; 11 Research Organization for Nano and Life Innovation, Waseda University, 2-2 Wakamatsu, Shinjuku-Ku, Tokyo, 162-8480 Japan

**Keywords:** Microbial interaction, Gut ecosystem, Pathobiont, Symbiont, *Fusobacterium*, *Faecalibacterium*

## Abstract

**Background:**

The human gut harbors a diverse microbiota that is crucial for maintaining health but also contributes to several diseases. Understanding how microbial communities are assembled and maintained is critical for advancing gut health.

**Results:**

We identified a unique interaction between the pathobiont *Fusobacterium varium* and the symbiont *Faecalibacterium prausnitzii*, both members of the gut microbial community; their interaction is driven by metabolites and direct cell-to-cell contact. Growth of *F. varium* was inhibited in the presence of *F. prausnitzii* because of a decrease in pH and an increase in *β*-hydroxybutyric acid. Conversely, the growth of *F. prausnitzii* was promoted in the presence of *F. varium*, likely via direct contact.

**Conclusions:**

These findings highlight the importance of metabolite-driven interactions and direct contact in shaping gut microbial communities and emphasize the potential of interactions between *F. prausnitzii* and *F. varium* in influencing gut health.

Video Abstract

**Supplementary Information:**

The online version contains supplementary material available at 10.1186/s40168-025-02168-w.

## Background

The human gut harbors an enormous density of microorganisms belonging to hundreds of different species, collectively known as the gut microbiota [1, 2]. Its taxonomic diversity is commonly profiled by sequencing the 16S ribosomal RNA genes. High-throughput genomic profiling approaches have revealed that the human gut microbiota are integral to the maintenance of health, and alterations to this community, known as dysbiosis, have been linked to numerous diseases [1, 2].

Although the loss of beneficial microbial species can exacerbate diseases, pathobionts have also been identified in the guts of patients with various diseases [3]; this term was introduced in 2008 in a study on *Helicobacter hepaticus* and was defined as a gut commensal bacterium with pathogenic potential [4].

*Fusobacterium* species (*Fusobacterium nucleatum*, *Fusobacterium necrophorum*, and *Fusobacterium varium*) are present in the oral cavity and are associated with infectious diseases, such as oropharyngeal infection and periodontal disease [5, 6]. These species also form part of the gut microbiota in humans. Although many *Fusobacterium* species are likely harmless or of uncertain health relevance in the gut, some damage the intestinal barrier and promote inflammation in intestinal diseases [7, 8]. For example, *F. nucleatum* is abundant in the intestinal tissues of patients with colorectal cancer [9, 10] and inflammatory bowel disease [11], whereas *F. varium* has been found in considerable numbers in patients with ulcerative colitis in Japan [12–14].

Increasing evidence suggests that the overgrowth of pathobionts is a risk factor for several diseases; therefore, it is critical to understand how a diverse gut microbial ecosystem is assembled and maintained. Two broad ecological interactions have been hypothesized to underlie the stability and resilience of the gut microbiota: competition and cooperation [15]. The symbiotic microbiota protects against pathogen colonization and the overgrowth of indigenous pathobionts [16]. These protective mechanisms are complex and include competitive microbial–microbial interactions and the induction of host immune responses. Bacteria can directly inhibit each other’s growth through spatial and nutritional competition, which includes factors such as physical location, oxygen concentration, pH, and nutrient availability. Bacteria can also produce inhibitory compounds, including bacteriocins and metabolic byproducts such as secondary bile acids and short-chain fatty acids [16].

Cooperation between microbiota members has also been discovered [15]. For example, an in vitro culture experiment combined with human microbiome analysis has demonstrated that dietary cross-feeding can fuel the overgrowth of pathobionts, including Enterobacteriaceae and Bacteroidales species, in the context of undernutrition [17].

In this study, we sought to gain new insights into the complex microbial interactions in the gut that contribute to defense against pathobiont *Fusobacterium* species. We identified an inverse correlation between *Fusobacterium* and *Faecalibacterium*, particularly between the pathobiont *F. varium* and the symbiont *Faecalibacterium prausnitzii*, within the gut microbial community of Japanese adults. Through bacterial culture experiments, we elucidated that metabolite-driven interactions and direct contact promote the growth of *F. prausnitzii* and inhibit that of *F. varium*.

## Methods

### Human samples

Participants collected their stool samples at home without any restrictions, such as fasting, and submitted them to the health examination site within 5 days. Body weight and height were measured, and information on diseases was obtained through health examinations. Fecal samples were preserved in guanidine thiocyanate solution (TechnoSuruga Laboratory, Shizuoka, Japan) as previously described [18], which allows for storage at room temperature.

### Microbiome analysis

DNA extraction and 16S rRNA gene amplicon sequencing were performed as previously described [19]. Briefly, the V3–V4 region of the 16S rRNA gene was amplified from fecal DNA and sequenced on an Illumina MiSeq platform with a Nextera XT Index Kit v2 Set A (Illumina, San Diego, CA, USA). The sequence reads were analyzed in the Quantitative Insights Into Microbial Ecology (QIIME) software package [20] and QIIME Analysis Automating Script (Auto-q) [21] with SILVA v128 reference sequence [22].

The same DNA was used for shotgun metagenomic sequencing performed for us by Takara Bio (Kusatsu, Japan). DNA was fragmented using a Covaris focused ultrasonicator (Covaris, Woburn, MA, USA) and 150-bp paired-end sequenced on a NovaSeq 6000 sequencing system (Illumina) with a ThruPLEX DNA-Seq Kit (Takara Bio). Raw sequence reads derived from the human genome were removed in Bowtie 2 (v2.2.5) [23] using the GRCh37 human reference genome (downloaded on December 2, 2021). Shotgun metagenomic sequencing data were analyzed by the Kraken 2 (v.2.0.9-beta)–Bracken (v.2.5.3) method [24, 25].

### Bacterial strains and cultures

*F. varium* (JCM 6320), *Faecalibacterium duncaniae* (JCM 31915, formerly *F. prausnitzii*, referred to in this study by the old name), *Phocaeicola vulgatus* (JCM 5826, formerly *Bacteroides vulgatus*, referred to in this study by the old name), *Blautia wexlerae* (JCM 31267), *Bifidobacterium longum* (JCM 1217), and *Akkermansia muciniphila* (JCM 33894) were obtained from the RIKEN BioResource Center (BRC) through the National BioResource Project of the MEXT/AMED, Japan.

All bacterial strains were cultured anaerobically in YCFA medium (no. 1130, https://www.jcm.riken.jp/cgi-bin/jcm/jcm_grmd?GRMD=1130) at 37 °C in a Bactron 300 anaerobic chamber (Toei Kaisha, Tokyo, Japan). For co-culture, individual strains were pre-cultured in YCFA for 1 day and diluted with fresh YCFA to an optical density at 600 nm of 0.3 (1–3 × 10^8^ cells/ml). A 0.1-ml aliquot of the diluted culture was inoculated into 5 ml of fresh YCFA medium and incubated statically under anaerobic conditions.

### Quantitative PCR to measure bacterial cell number

DNA was extracted from bacterial cultures using the bead-beating method as previously described with slight modifications [26]. Briefly, 0.2 ml of culture was added to a 2-ml vial (WakenBtech, Tokyo, Japan) containing 0.3 ml of lysis buffer (No. 10, Kurabo Industries, Osaka, Japan) and 0.5 g of 0.1-mm glass beads. The cells were mechanically disrupted by bead beating in a Cell Destroyer PS1000 (Bio Medical Science, Tokyo, Japan) at 4260 rpm for 50 s at room temperature (25 °C). The mixture was centrifuged at 13,000 × *g* for 5 min at room temperature, and DNA was extracted from 0.2 ml of the supernatant using an automatic nucleic acid extraction system (Gene Prep Star PI-80X, Kurabo Industries).

Quantitative PCR was performed in the CFX Opus 96 Real-Time PCR system (Bio-Rad Laboratories, Hercules, CA, USA) with the Real-Time PCR Detection Kit (TechnoSuruga Laboratory) and TB Green Premix Ex Taq II (Takara Bio), in accordance with the manufacturers’ instructions. Bacterial cell number was calculated from the DNA copy number.

### LC–MS/MS

LC–MS/MS metabolome analysis was performed as previously described [26]. Bacterial culture was centrifuged at 15,000 × *g* at 4 °C for 10 min, and the supernatant was filtered through a Millex 0.22-µm PVDF syringe filter (Merk, Rahway, NJ, USA). The pellet was washed with 1 ml of PBS and suspended in 100 µl of PBS.

For the analysis of primary metabolites, the filtered supernatant (100 µl) or bacterial cell suspension (100 µl) was diluted with water (100 µl; Fujifilm Wako Pure Chemical, Osaka, Japan) and mixed with 400 µl of methanol (Fujifilm Wako Pure Chemical) containing 2-morpholinoethanesulfonic acid (Fujifilm Wako Pure Chemical) as an internal standard and then with 400 µl of chloroform (Nacalai Tesque, Kyoto, Japan). Samples were centrifuged at 20,000 × *g* at 4 °C for 15 min, and 200 µl of the aqueous layer was centrifugally filtered through a 5-kDa cutoff filter (Human Metabolome Technologies, Tokyo, Japan). The filtrate was lyophilized, resuspended in ultrapure water (Fujifilm Wako Pure Chemical), and analyzed by LC–MS/MS in a Nexera system (Shimadzu GLC, Tokyo, Japan) equipped with two LC-40D pumps, a DGU-405 degasser, a SIL-40C autosampler, a CTO-40C column oven, and a CBM-40 control module, coupled to an LCMS-8050 triple-quadrupole mass spectrometer (Shimadzu). A pentafluorophenylpropyl column (Discovery HS F5, 150 mm × 2.1 mm, 3 µm; Sigma-Aldrich) was used to separate metabolites. Instrument control and data analysis were performed in LabSolutions LCMS software with the LC/MS/MS Method Package for Primary Metabolites, ver. 2 (Shimadzu).

For the analysis of short-chain fatty acids, the filtered supernatant (10 µl) was mixed with 40-µl ethanol containing 2-ethylbutyric acid (Fujifilm Wako Pure Chemical) as an internal standard and centrifuged at 20,000 × *g* at 4 °C for 10 min. For derivatization, the supernatant was mixed with 3-nitrophenylhydrazine, 1-ethyl-3-carbodiimide, and pyrimidine and incubated for 30 min at 25 °C. The sample was diluted with 75% methanol containing 0.5% formic acid and analyzed by LC–MS/MS in the Nexera system described above. A Mastro C18 column (150 mm × 2.0 mm, 3 µm; Shimadzu) was used to separate fatty acids. Instrument control and data analysis were performed in LabSolutions LCMS software with the LC/MS/MS Method Package for Short-Chain Fatty Acids (Shimadzu).

### Whole-transcriptome RNA-seq

Bacterial cells were collected by centrifugation at 10,000 × *g* for 5 min at 4 °C. Total RNA was isolated using a NucleoSpin RNA kit (Takara Bio) and sent to Takara Bio for RNA-seq analysis. Briefly, RNA was quantified using a NanoDrop spectrophotometer (Thermo Fisher Scientific, Tokyo, Japan), and its size distribution was assessed by using an Agilent 2200 TapeStation system (Agilent Technologies, Santa Clara, CA, USA). Ribosomal RNA was depleted, and a cDNA library was constructed by using an Agilent XT-Auto system (Agilent Technologies) and Biomek i7 (Beckman Coulter, Tokyo, Japan) with Illumina Stranded Total RNA Prep, ligation with Ribo-Zero Plus kit (Illumina) and IDT for Illumina RNA UD indexes, ligation (Illumina) in accordance with the Illumina Stranded Total RNA Prep, and ligation with Ribo-Zero Plus reference guide v02.

Samples were sequenced for us by Takara Bio using 150-bp paired-end sequencing on a NovaSeq 6000 system with a NovaSeq 6000 S4 Reagent Kit v1.5 and a NovaSeq Xp 4-Line Kit v1.5 (both from Illumina) in accordance with the NovaSeq 6000 Sequencing System guide v16 and the guide for bcl2fastq2 conversion software v2.20. RNA-seq reads were mapped to the *F. varium* and *F. duncaniae* (formerly *F. prausnitzii*) reference genomes (accession nos. GCF_900637705.1 and GCF_010509575.1), and transcript abundance was evaluated as transcripts per million per genomic element using a DRAGEN Bio-IT Platform v3.9.3 (Illumina).

### Scanning electron microscopy

Bacterial cells were fixed by mixing the cultures (1:1) with 2% glutaraldehyde in 0.1-M phosphate buffer. The samples were sent to Hanaichi UltraStructure Research Institute and examined under a scanning electron microscope (JSM-7500F, JEOL, Tokyo, Japan).

### Statistical analyses

Statistical analyses were performed in GraphPad Prism 8 (GraphPad Software, La Jolla, CA, USA). For correlation analysis, Pearson and Spearman correlations were calculated. To assess statistical significance, one-way or two-way ANOVA was used for multiple-group comparisons, and the Mann–Whitney *U*-test or unpaired *t*-test was used for two-group comparisons. A *P*-value less than 0.05 was considered significant.

## Results

### Gut microbiome composition and the inverse relationship between *F. varium* and *F. prausnitzii* in Japanese adults

**Fig. 1:**
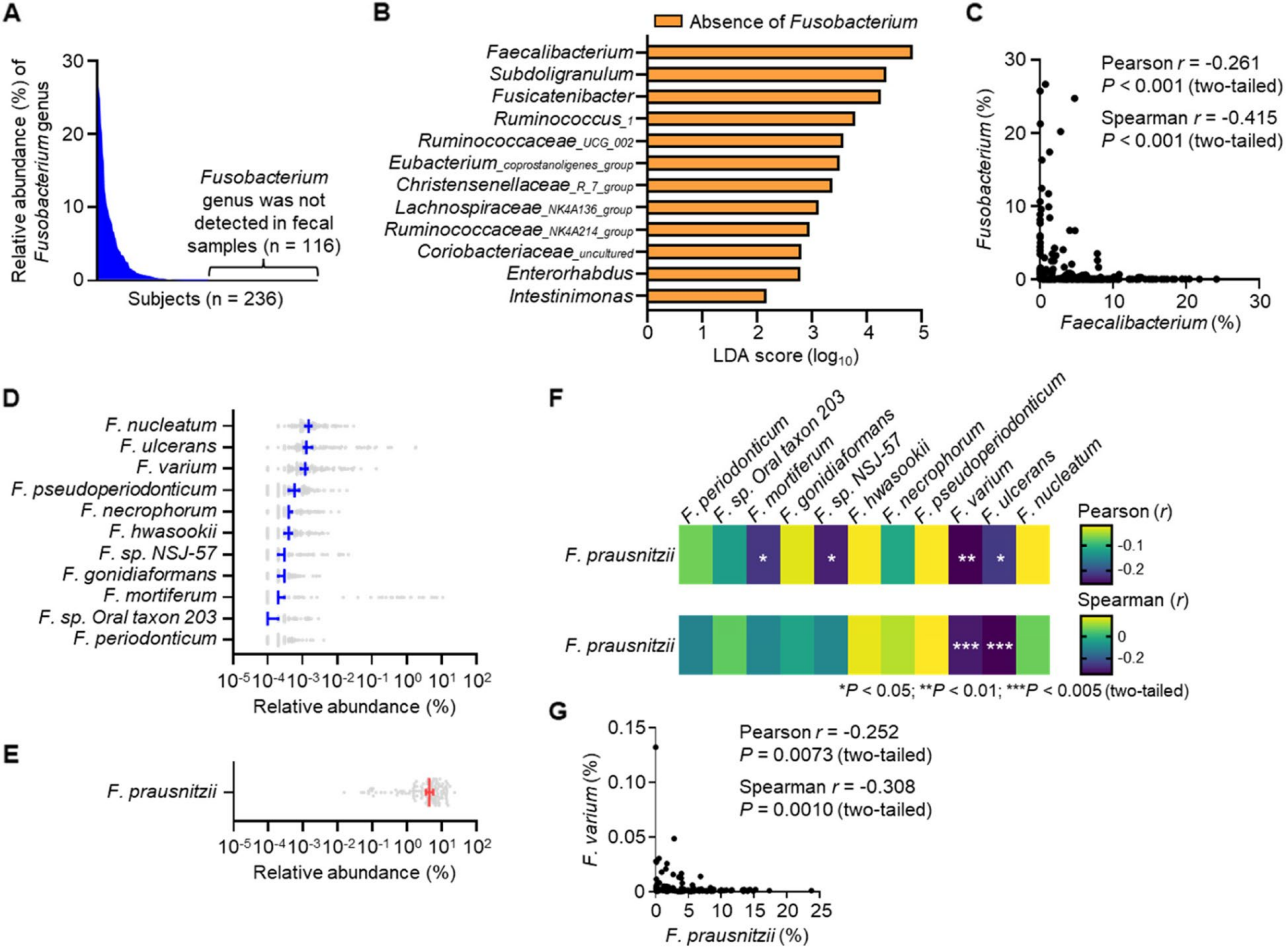
Inverse relationship between *Fusobacterium* and *Faecalibacterium* in Japanese adults. **A** Distribution of the relative abundance of the *Fusobacterium* genus in the feces of 236 participants (Supplementary Table 1). **B** Differences in bacterial taxonomy ranked by the linear discriminant analysis (LDA) effect size (*P* < 0.05) among participants with (*n* = 120) and without (*n* = 116) *Fusobacterium* in their feces. **C** Scatterplots of Pearson and Spearman correlation analysis between the relative abundances of the *Fusobacterium* and *Faecalibacterium* genera (*n* = 236). **D**, **E** Relative abundance of **D** *Fusobacterium* and **E** *Faecalibacterium* species in the 112 participants. **F** Heatmaps of Pearson and Spearman correlation analysis between the relative abundances of *Fusobacterium* and *Faecalibacterium* species (*n* = 112). **G** Scatterplots of Pearson and Spearman correlation analysis between the relative abundances of *F. varium* and* F. prausnitzii* (*n* = 112). The data were obtained by **A**, **B**, **C** 16S rRNA gene amplicon sequencing or **D**, **E**, **F**, **G** shotgun metagenomic sequencing

In our previous cross-sectional study, we profiled the bacterial composition of the gut microbiota in subjects lacking *Fusobacterium* in their feces by using 16S amplicon sequencing data from 236 Japanese participants (Supplementary Table 1) [19]. The *Fusobacterium* genus was detected in 120 participants and was absent in 116 (Fig. 1A and Supplementary Table 1).

Linear discriminant analysis effect size [27] was applied to rank the genera of intestinal bacteria that differed between participants with and without *Fusobacterium* in their feces (Fig. 1B). *Faecalibacterium* showed the highest linear discriminant analysis score, prompting further investigation of the relationship between the fecal abundances of the *Faecalibacterium* and *Fusobacterium* genera. A scatterplot revealed an inverse correlation between their abundances (Fig. 1C).

To investigate this relationship at the species level, we performed shotgun sequencing of the gut microbiota of fecal samples from 112 Japanese participants and identified 11 species of *Fusobacterium* and 1 species of *Faecalibacterium* (Fig. 1D, E). Several *Fusobacterium* species were negatively correlated to *F. prausnitzii*, with *F. varium* showing a significant correlation (*P* < 0.01) in both Pearson and Spearman correlation analyses (Fig. 1F). A scatterplot further confirmed the negative correlation between *F. varium* and *F. prausnitzii* (Fig. 1G).

Given the potential beneficial roles of *F. prausnitzii* in maintaining human gut health [28], we focused on the interaction between the potentially pathogenic *F. varium* and the symbiotic *F. prausnitzii*.

### Suppression of *F. varium* growth by *F. prausnitzii* and role of pH and β-hydroxybutyric acid

**Fig. 2:**
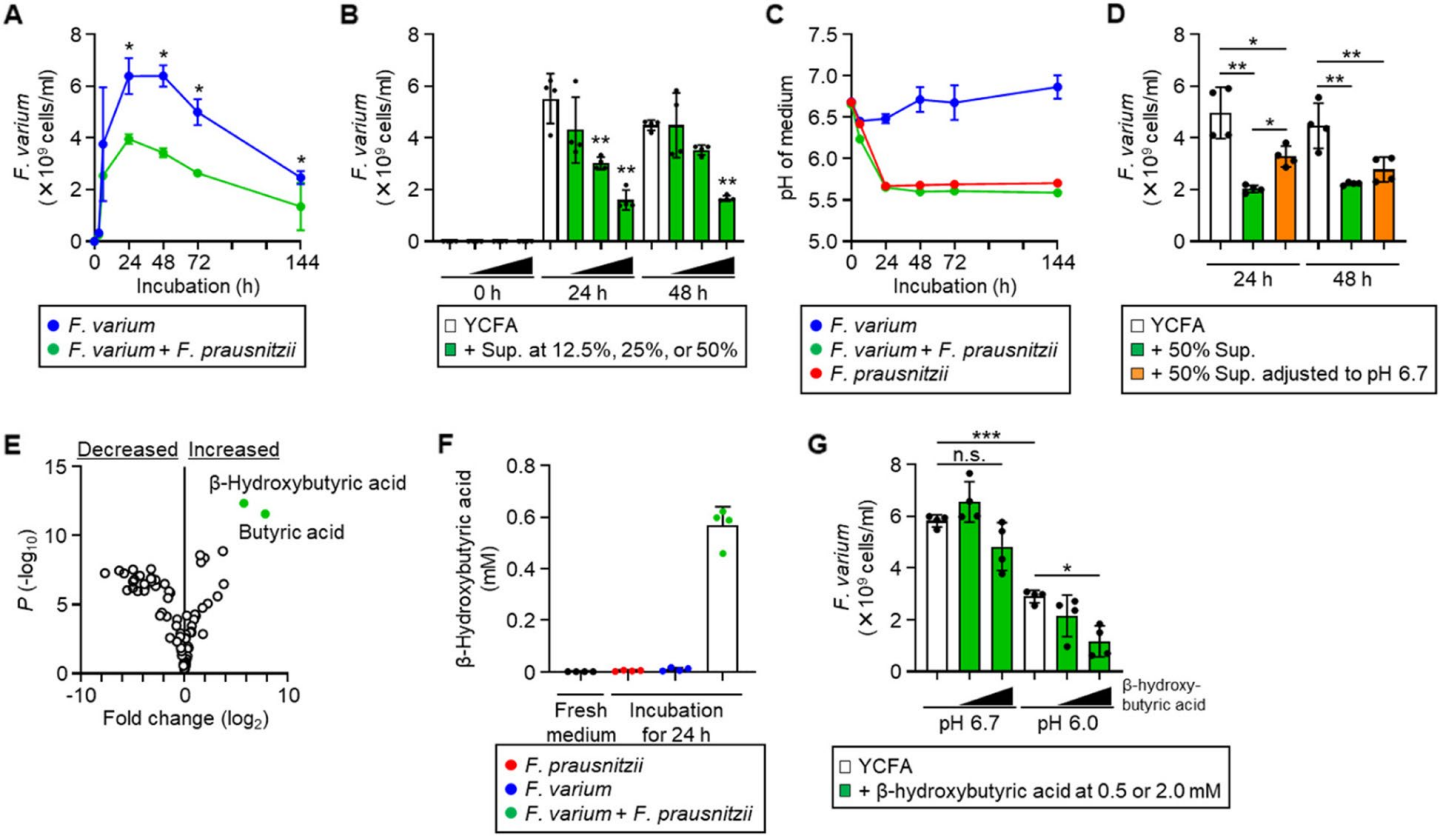
Suppression of *F. varium* growth by *F. prausnitzii* via low pH and *β*-hydroxybutyric acid. **A** Effect of *F. prausnitzii* on *F. varium* growth. *F. varium* was cultured in the presence or absence of *F. prausnitzii*. *P < 0.05 (two-tailed Mann–Whitney *U*-test). **B** Effect of the supernatant from co-culture of *F. varium* and *F. prausnitzii* on *F. varium* growth. *F. varium* was cultured in the absence or presence of the supernatant at the indicated concentrations. **P < 0.01 (one-way ANOVA). **C** The pH of culture medium during bacterial growth. *F. varium* alone, *F. prausnitzii* alone, or both were cultured (*n* = 4, mean ± 1 SD). **D** Impact of pH on the inhibitory effect of co-culture supernatant. *F. varium* was cultured in the absence or presence of the supernatant from *F. varium* and *F. prausnitzii* co-culture adjusted or not to pH 6.7 (the initial pH of the medium). *P < 0.05, **P < 0.01 (one-way ANOVA). **E** Volcano plot of bacterial metabolites. *F. varium* and *F. prausnitzii* were co-cultured for 24 h, and the primary metabolites and short-chain fatty acids were measured by LC–MS/MS. Green dots indicate metabolites that increased by > 10 × in the co-culture supernatant compared with fresh medium (*n* = 4). Statistical significance was evaluated by using two-tailed unpaired *t*-test. **F** *β*-Hydroxybutyric acid concentration in bacterial cultures. *F. prausnitzii* alone, *F. varium* alone, or both were cultured for 24 h. The concentration of *β*-hydroxybutyric acid was measured by LC–MS/MS (*n* = 4, mean ± 1 SD). **G** Effects of pH and *β*-hydroxybutyric acid on *F. varium* growth. *F. varium* was cultured for 24 h in YCFA (pH 6.7 or 6.0) in the absence or presence of *β*-hydroxybutyric acid. *P < 0.05, ***P < 0.001; n.s., not significant (one-way ANOVA). **A**, **B**, **D**, **G** The number of *F. varium* cells was assessed by quantitative PCR (*n* = 4, mean ± 1 SD)

To investigate bacterial interactions that may suppress the growth of *F. varium* in vitro, we cultured it alone or with *F. prausnitzii*. In our culture setup, *F. varium* began to proliferate within 3 h of inoculation, with cell number peaking at 24–48 h and then gradually decreasing (Fig. 2A). In the presence of *F. prausnitzii*, the growth of *F. varium* was significantly suppressed (Fig. 2A). Supplementation with the supernatant from *F. prausnitzii* and *F. varium* co-culture inhibited *F. varium* growth in a dose-dependent manner (Fig. 2B). These findings suggest that *F. prausnitzii* creates an environment that suppresses *F. varium* growth.

Given that some intestinal bacteria, including *F. prausnitzii*, maintain a low pH in the gut to secure their ecological niche [28], we measured the pH of the medium during bacterial culture. In the presence of *F. varium* alone, the pH remained neutral throughout the culture period (Fig. 2C). In contrast, the pH decreased to approximately pH 5.7 in *F. prausnitzii* culture regardless of the presence of *F. varium* (Fig. 2C).

To assess the role of low pH in the inhibitory effect of *F. prausnitzii* on *F. varium* growth, we adjusted the pH of the co-culture supernatant to 6.7 (the initial pH of the medium). The inhibitory effect of this supernatant on *F. varium* growth was lower than that of the non-neutralized co-culture supernatant, although it was not completely abolished (Fig. 2D). These results suggest that low pH is important for the suppression of *F. varium*, although additional factors are involved.

To identify these unknown factors, we analyzed the metabolome by LC–MS/MS of the co-culture supernatant. A volcano plot revealed significant changes in metabolite profiles and identified two metabolites that increased more than 10 × in the co-culture supernatant in comparison with the fresh medium (Fig. 2E). One of them, butyric acid, was produced by both *F. prausnitzii* and *F. varium* (Supplementary Fig. 1), but the other one, β-hydroxybutyric acid, was produced only in the co-culture of *F. prausnitzii* and *F. varium* (Fig. 2F), and its production was consistent with the inhibitory effects on *F. varium* growth (Fig. 2B and Supplementary Fig. 2). Low pH and *β*-hydroxybutyric acid had an additive inhibitory effect on *F. varium* growth (Fig. 2G). Taken together, these findings suggest that *F. prausnitzii* creates an environment unfavorable to *F. varium* growth, characterized by a reduced pH and increased concentration of *β*-hydroxybutyric acid.

### Metabolic and gene expression changes induced in *F. varium* by low pH and high β-hydroxybutyric acid concentration

**Fig. 3:**
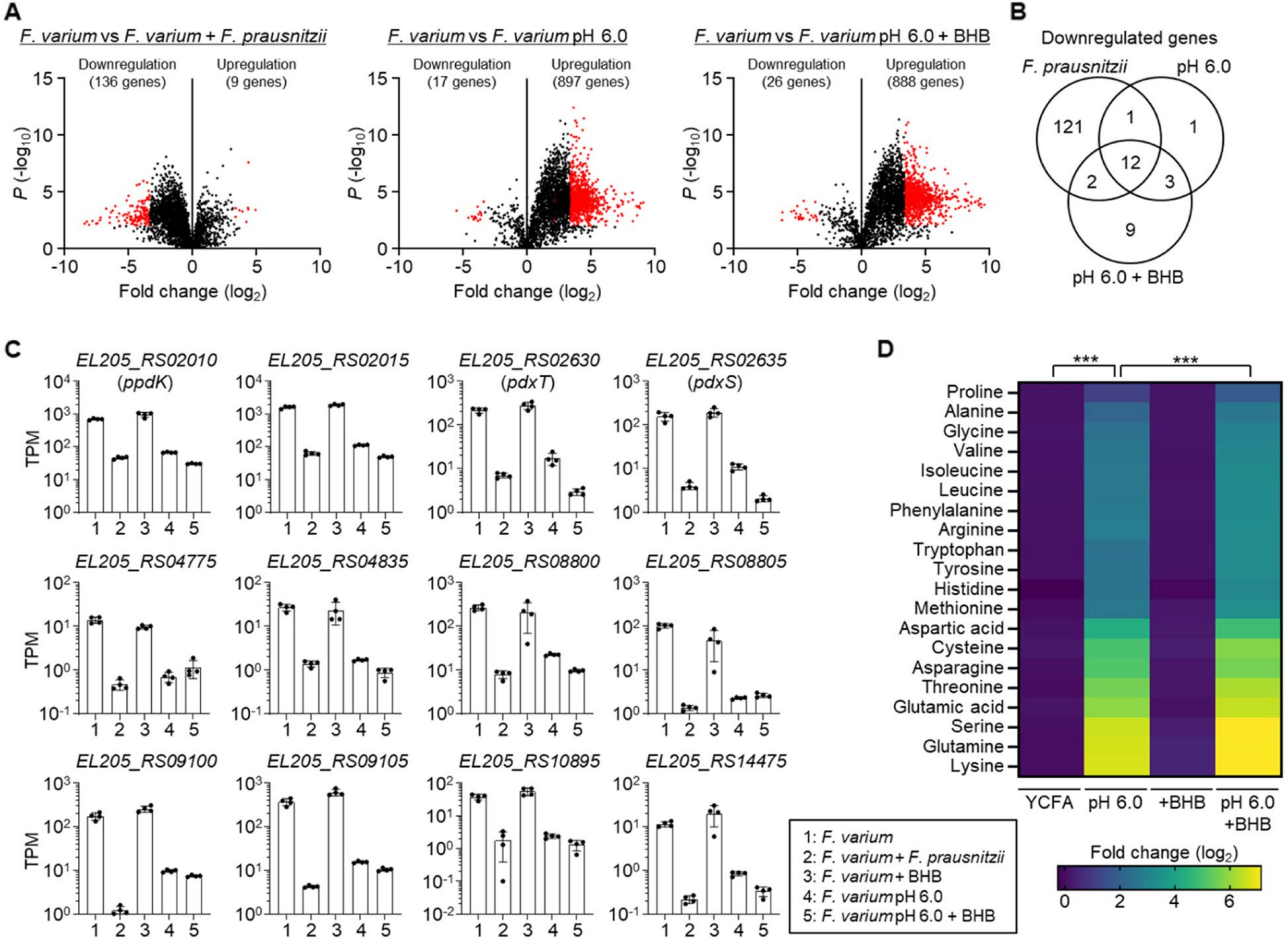
Gene expression and metabolome analyses of *F. varium* cultured in low-pH and high-*β*-hydroxybutyric acid conditions. **A** Volcano plots of gene expression. *F. varium* was cultured for 24 h in YCFA, YCFA in the presence of *F. prausnitzii*, YCFA (pH 6.0), or YCFA (pH 6.0) supplemented with 2-mM *β*-hydroxybutyric acid (BHB). Gene expression was analyzed by RNA-seq (*n* = 4). Red dots indicate genes upregulated or downregulated by > 10 × (*P* < 0.01, two-tailed unpaired *t*-test). **B** Venn diagram of genes downregulated under the conditions used in **A**. **C** Transcript levels of the 12 genes shown in **B** that were consistently downregulated under all 3 conditions. TPM, transcripts per million. **D** Heatmap of amino acid contents. *F. varium* was cultured for 24 h in YCFA, YCFA (pH 6.0), YCFA supplemented with 2-mM *β*-hydroxybutyric acid (BHB), or YCFA (pH 6.0) supplemented with 2-mM BHB. Intracellular amino acid contents were measured by LC–MS/MS (*n* = 4) and normalized by bacterial cell number assessed by quantitative PCR. ***P < 0.001 (two-way ANOVA)

RNA-seq analysis revealed that many *F. varium* genes were differentially expressed when it was exposed to *F. prausnitzii*, low pH, or a combination of *β*-hydroxybutyric acid and low pH (Fig. 3A). Among the genes that were downregulated by > 10 × (*P* < 0.01), 12 genes were downregulated consistently under all these conditions (Fig. 3B). The functions of nine of these genes are unknown, whereas three are annotated: *EL205_RS02010* (gene name: *ppdK*), *EL205_RS02630* (*pdxT*), and *EL205_RS02635* (*pdxS*). The expression patterns of these three genes were consistent with the inhibitory effect on *F. varium* growth (Fig. 3C).

The enzymes PdxT and PdxS are involved in pyridoxal 5′-phosphate (PLP) synthesis [29] and amino acid metabolism [30, 31], and *F. varium* is known to use amino acids such as glutamic acid, histidine, lysine, and serine for growth [32]. LC–MS/MS analysis revealed that lysine, glutamine, serine, and glutamic acid accumulated in *F. varium* cells grown at low pH, and their accumulation was more pronounced when *β*-hydroxybutyric acid was also present (Fig. 3D). The glutaminase PdxT hydrolyzes glutamine to produce PLP [29], so the observed accumulation of glutamine aligns with the decreased expression of *pdxT*. Collectively, these findings suggest that a combination of low pH and increased *β*-hydroxybutyric acid disrupts the metabolism of *F. varium*, including amino acid catabolism, which contributes to the inhibition of its growth in the presence of *F. prausnitzii*.

### Broad inhibitory effects of intestinal bacteria on *F. varium* growth

**Fig. 4:**
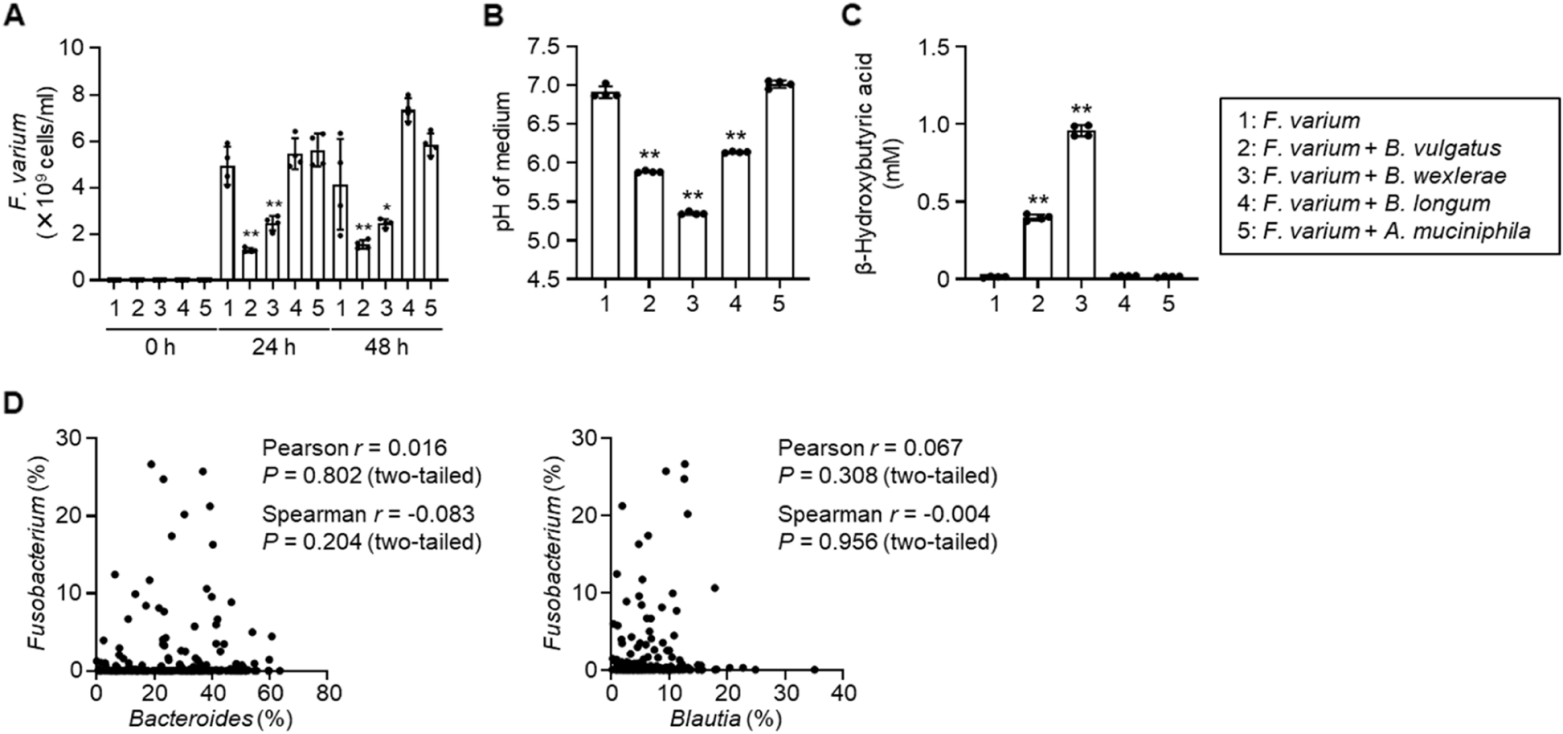
Inhibition of *F. varium* growth by other intestinal bacteria. **A** Effects of intestinal bacterial species on *F. varium* growth. *F. varium* was cultured in the absence or presence of *B. vulgatus* (now named *P. vulgatus*), *B. wexlerae*, *B. longum*, or *A. muciniphila*. The number of *F. varium* cells was assessed by quantitative PCR (*n* = 4, mean ± 1 SD). *P < 0.05, **P < 0.01 (one-way ANOVA). **B** The pH of bacterial cultures. *F. varium* was cultured for 24 h in the absence or presence of the above species (*n* = 4, mean ± 1 SD). **P < 0.01 (one-way ANOVA). **C** Concentration of *β*-hydroxybutyric acid in bacterial cultures. *F. varium* was cultured as in **B**. The concentration of *β*-hydroxybutyric acid was measured by LC–MS/MS (*n* = 4, mean ± 1 SD). **P < 0.01 (one-way ANOVA). **D** Scatterplots for Pearson and Spearman correlation analysis between the relative abundances of *Fusobacterium* and *Bacteroides* or *Blautia* assessed by 16S rRNA gene amplicon sequencing analysis of Japanese adults (*n* = 236) (Supplementary Table 1)

Since pH control and *β*-hydroxybutyric acid production are not exclusive to *F. prausnitzii* [28, 33], we examined the inhibitory effects of other bacterial species predominant in the human gut [34]. The growth of *F. varium* was suppressed by co-culturing with *B. vulgatus* (now known as *P. vulgatus* but referred to in this study by the old name) or *B. wexlerae*, but not with *B. longum* or *A. muciniphila* (Fig. 4A). Notably, the inhibitory effect in these co-cultures matched the decrease in pH and increase in *β*-hydroxybutyric acid concentration (Fig. 4B, C). These results suggest that multiple intestinal bacterial species can inhibit the growth of *F. varium*, and the combination of low pH and increased *β*-hydroxybutyric acid likely contributes to suppressing *F. varium* overgrowth in the gut.

However, the abundance of *Fusobacterium* was not correlated with that of *Bacteroides* or *Blautia* in the Japanese microbiome data (Fig. 4D), suggesting that there may be other specific microbial interaction mechanisms unique to *F. prausnitzii*.

### Promotion of *F. prausnitzii* growth by *F. varium* and the role of direct bacterial contact

**Fig. 5:**
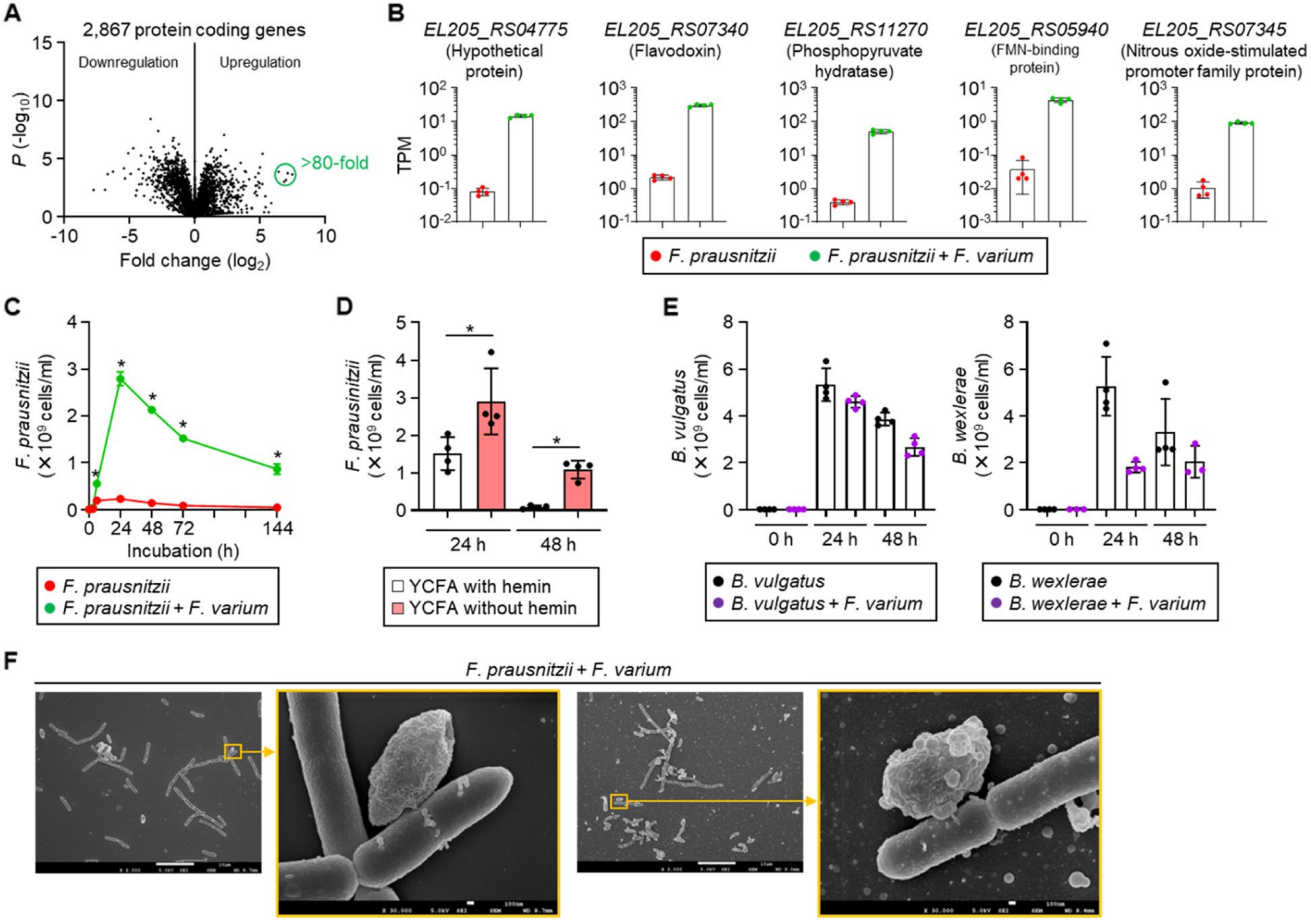
Promotion of *F. prausnitzii* growth by *F. varium*. **A** Volcano plot of gene expression in *F. prausnitzii*. *F. prausnitzii* was cultured for 24 h in YCFA in the presence or absence of *F. varium*. Gene expression was analyzed by RNA-seq (*n* = 4). Green circle indicates genes upregulated by > 80 × (*P* < 0.01, two-tailed unpaired *t*-test). **B** Transcript levels of the five genes upregulated > 80 × in **A**. TPM, transcripts per million. **C** Effect of *F. varium* on *F. prausnitzii* growth. *P < 0.05 (two-tailed Mann–Whitney *U*-test). **D** Effects of iron availability on *F. prausnitzii* growth. *F. prausnitzii* was cultured anaerobically in YCFA supplemented or not with hemin. *P < 0.05 (two-tailed Mann–Whitney *U*-test). **E** Effects of *F. varium* on growth of *B. vulgatus* and *B. wexlerae* cultured anaerobically in YCFA. **F** Scanning electron microscopy images of *F. prausnitzii* and *F. varium*. Bacteria were co-cultured anaerobically in YCFA for 24 h, fixed with glutaraldehyde, and observed under a scanning electron microscope (JSM-7500F, JEOL, Tokyo, Japan). Scale bars: 10 μm or 100 nm. **C**, **D**, **E** The number of bacterial cells was assessed by quantitative PCR (*n* = 4, mean ± 1 SD)

To investigate the functional changes in *F. prausnitzii* in the presence of *F. varium*, we performed a comprehensive gene expression analysis. RNA-seq revealed that many genes in *F. prausnitzii* were differentially expressed when it was exposed to *F. varium* (Fig. 5A). Among these, five genes were dramatically upregulated by > 80 × (*P* < 0.01); the functions of four of them are known (Fig. 5B). Flavodoxin is an electron-transfer protein involved in various metabolic reactions during bacterial growth; it functions through the flavin mononucleotide (FMN) cofactor. The synthesis of flavodoxin is induced under stress conditions, such as low iron levels, to help bacteria adapt to environmental changes [35, 36].

The growth of *F. prausnitzii* was promoted by *F. varium* (Fig. 5C) and by a medium with no hemin supplementation (Fig. 5D), but *F. varium* did not promote and even tended to suppress the growth of *B. vulgatus* and *B. wexlerae* (Fig. 5E). These findings suggest that the inverse correlation between the abundances of *F. varium* and *F. prausnitzii* in the human gut (Fig. 1C) may result from both the suppression of *F. varium* growth and the promotion of *F. prausnitzii* growth.

Unexpectedly, supplementation with the co-culture supernatant did not affect the cell number of *F. prausnitzii* (Supplementary Fig. 3 A). To further investigate the effect of *F. varium*’s secreted products, we co-cultured it with *F. prausnitzii* in a chamber where the two species were separated by a filter; we confirmed that the filter was impermeable to the cells. In these settings, *F. varium* had no effect on the growth of *F. prausnitzii* (Supplementary Fig. 3B).

Since direct cell-to-cell bacterial communication has been reported [37, 38], we examined bacterial cells in the co-culture under a scanning electron microscope. The cells of *F. prausnitzii* and *F. varium* had distinct shapes and surfaces (Supplementary Fig. 4) and appeared to adhere to each other (Fig. 5F). Our results suggest that the growth of *F. prausnitzii* was likely promoted by direct contact with *F. varium*, although the mechanism through which this direct connection promotes growth remains unclear.

## Discussion

Our findings in this study suggest that *F. varium*, a potential pathobiont, and *F. prausnitzii*, a beneficial symbiont, interact with each other within the human gut both directly and indirectly. Growth of *F. varium* was suppressed in the presence of *F. prausnitzii*, likely because of acidification and *β*-hydroxybutyric acid accumulation. Conversely, *F. prausnitzii* appeared to benefit from the presence of *F. varium*, potentially through direct cell-to-cell interactions. These observations highlight the complexity of microbial interactions that maintain gut homeostasis and provide new insights into symbiont–pathobiont dynamics in gut health.

Our in vitro culture experiments suggest that the relationship between *F*. *prausnitzii* and *F. varium* is not merely a result of competition. If their relationship was purely competitive, *Blautia* and *Bacteroides* would also be expected to show an inverse correlation with *Fusobacterium*, but no such correlation was observed in human data. Unlike the growth of *B. wexlerae* and *B. vulgatus*, that of *F. prausnitzii* was promoted through interaction with *F. varium*. This response may enhance the inhibitory effect of *F. prausnitzii* on *F. varium*, ultimately leading to the inverse relationship between their abundances in the human gut microbial community.

Our results indicate that *F. prausnitzii* suppresses *F. varium* growth through acidification- and metabolite-mediated mechanisms. The production of short-chain fatty acids such as butyrate and its derivatives, including *β*-hydroxybutyric acid, is a well-known mechanism driving acidification of the gut environment [39, 40]. Acidification creates an inhospitable environment for certain pathobionts, including *F. varium*, which thrive at neutral or slightly alkaline conditions. Our findings align with those of studies demonstrating that a low pH supports the growth of beneficial gut microbiota, thereby contributing to gut health [39–41].

In mammals, *β*-hydroxybutyric acid, a product of fatty acid oxidation, is an important regulator of gene expression, lipid metabolism, neuronal function, and overall metabolic rate [33]. In bacteria, it primarily serves as a substrate for the synthesis of polyhydroxybutyrate. Although the mechanisms remain poorly understood, *β*-hydroxybutyric acid affects gut microbiota composition and directly inhibits bacterial growth [42–44].

Our data suggest that *F. prausnitzii* benefits from *F. varium*, indicating a potential facilitative interaction. A plausible explanation is that *F. varium* produces metabolites or degrades substrates inaccessible to *F. prausnitzii* and thus produces nutrients that *F. prausnitzii* can use, suggesting a cross-feeding relationship similar to interactions between *Faecalibacterium* and *Bifidobacterium* [45]. However, our co-culture experiments suggest that these interactions may extend beyond metabolite-mediated communication and include direct cell-to-cell communication.

In direct bacterial communication, bacteria exchange signals, materials, or information through either physical contact or molecular mechanisms. This exchange occurs via structures such as nanotubes, type VI secretion systems, and membrane vesicles [37, 38, 46]. This communication is crucial in coordinating group behaviors, adapting to environmental changes, and mediating competition or cooperation within bacterial communities. Although the underlying mechanisms remain unclear, direct interaction between *F. prausnitzii* and *F. varium* may contribute to the stability of the gut microbiota and to overall gut health.

From a clinical perspective, *F. prausnitzii* is known for its beneficial effects on gut health, including its anti-inflammatory properties and role in maintaining intestinal barrier integrity, whereas *F. varium* has been implicated in diseases such as ulcerative colitis. Our study sheds light on the microbial dynamics underlying dysbiosis-related diseases. Since the suppression of *F. varium* may reduce disease risk and promote gut health, our findings suggest that enhancing the growth of *F. prausnitzii* could provide a novel therapeutic approach for preventing dysbiosis-related conditions.

Other intestinal bacteria, namely *B. vulgatus* and *B. wexlerae*, also suppressed the growth of *F. varium*. This observation highlights the intricate and competitive nature of microbial interactions. Further elucidation of the underlying molecular mechanisms may provide valuable insights into how specific bacterial species can be harnessed for healthcare and therapeutic applications.

This study has some limitations. The bacterial culture conditions used do not fully replicate the complexity of the human gut environment. The interactions reported here were observed under controlled conditions, and the influence of host factors, such as immune responses and diets, on microbial behavior remains unclear. Additionally, our findings are based on a cohort of Japanese adults and may not be generalizable to other populations or disease contexts. Future research should aim to validate these findings in diverse populations and investigate the in vivo implications of these interactions in both health and disease.

## Conclusions

Our findings provide new insights into the metabolic and ecological interactions shaping gut microbial dynamics. By elucidating the interplay between *F. varium* and *F. prausnitzii*, we highlight the crucial role of symbiont–pathobiont relationships in gut health. Continued research into the microbial interactions in the human gut will be essential for unraveling the complexities of gut dysbiosis and its role in disease, paving the way for novel microbiota-based therapies.

## Supplementary Information

Supplementary Material 1: Supplementary Table 1. Participant information Supplementary figures. Figure 1 Butyric acid concentration in *F. prausnitzii *and *F. varium* cultures. Each species alone or both together were cultured for 24 h. Butyric acid was measured by LC-MS/MS (*n* = 4, mean ± 1 SD). Supplementary Figure 2 Effect of supernatant from *F. prausnitzii *culture on *F. varium *growth. *Fusobacterium varium *was cultured in the absence or presence of the supernatant from *F. prausnitzii* culture at a concentration of 12.5%, 25%, or 50%. The number of*F. varium *cells was measured by quantitative PCR (*n* = 4, mean ± 1 SD).**P < 0.01 (one-way ANOVA). Supplementary Figure 3 Effect of products secreted by *F. varium *on *F. prausnitzii *growth. (A) Effect of the supernatant from co-culture of *F. prausnitzii *and *F. varium *on *F. prausnitzii *growth. *Faecalibacterium prausnitzii *was cultured in the absence or presence of the supernatant at a concentration of 12.5%, 25%, or 50%. (B) Co-culture of *F. prausnitzii *and *F. varium*. The bacteria were co-cultured in a UniWells horizontal co-culture plate (Fujifilm Wako Pure Chemical, Osaka, Japan), in which the two species are co-cultured together on one side of the plate (closed) or are separated by a filter (pore size: 0.03 μm or 0.6 μm). In both panels, the number of *F. prausnitzii *cells was measured by quantitative PCR (*n* = 4, mean ± 1 SD). Supplementary Figure 4 Scanning electron microscopy images of *F. prausnitzii *and *F. varium*. Each species was cultured for 24 h; the cultures were fixed with glutaraldehyde and observed under a scanning electron microscope (JSM-7500F, JEOL, Tokyo, Japan). Scale bars, 1 μm.

## Data Availability

DNA sequencing data have been deposited in the DNA Databank of Japan (DDBJ) under the BioProject numbers PRJDB15701 (16S amplicon sequencing), PRJDB18854 (shotgun sequencing), and PRJDB18861 (RNA-seq).
