# Supplementary material for: Metabolite-mediated interactions and direct contact between Fusobacterium varium and Faecalibacterium prausnitzii
Source: Microbiome. 2025 Jul 28;13:175. doi: 10.1186/s40168-025-02168-w (PMC12302451; doi:10.1186/s40168-025-02168-w)
Supplement: Supplementary file 2 — Supplementary Material 1: Supplementary Table 1. Participant information Supplementary figures. Figure 1 Butyric acid concentration in F. prausnitzii and F. varium cultures. Each species alone or both together were cultured for 24 h. Butyric acid was measured by LC-MS/MS (n = 4, mean ± 1 SD). Supplementary Figure 2 Effect of supernatant from F. prausnitzii culture on F. varium growth. Fusobacterium varium was cultured in the absence or presence of the supernatant from F. prausnitzii culture at a concentration of 12.5%, 25%, or 50%. The number ofF. varium cells was measured by quantitative PCR (n = 4, mean ± 1 SD).**P < 0.01 (one-way ANOVA). Supplementary Figure 3 Effect of products secreted by F. varium on F. prausnitzii growth. (A) Effect of the supernatant from co-culture of F. prausnitzii and F. varium on F. prausnitzii growth. Faecalibacterium prausnitzii was cultured in the absence or presence of the supernatant at a concentration of 12.5%, 25%, or 50%. (B) Co-culture of F. prausnitzii and F. varium. The bacteria were co-cultured in a UniWells horizontal co-culture plate (Fujifilm Wako Pure Chemical, Osaka, Japan), in which the two species are co-cultured together on one side of the plate (closed) or are separated by a filter (pore size: 0.03 μm or 0.6 μm). In both panels, the number of F. prausnitzii cells was measured by quantitative PCR (n = 4, mean ± 1 SD). Supplementary Figure 4 Scanning electron microscopy images of F. prausnitzii and F. varium. Each species was cultured for 24 h; the cultures were fixed with glutaraldehyde and observed under a scanning electron microscope (JSM-7500F, JEOL, Tokyo, Japan). Scale bars, 1 μm. [file 40168_2025_2168_MOESM1_ESM.pptx]

## Slide 1
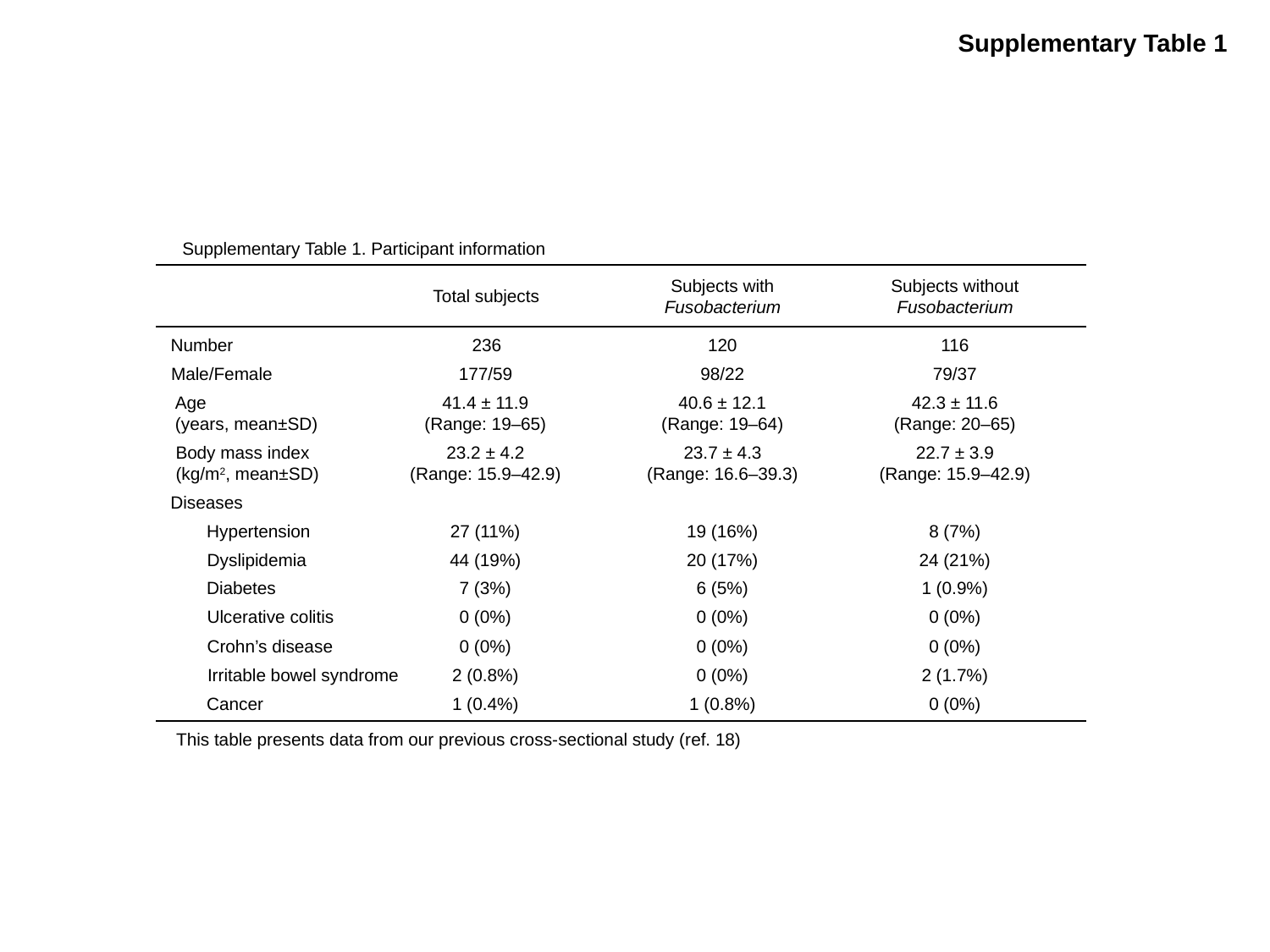

Supplementary Table 1
Supplementary Table 1. Participant information
Subjects with
Fusobacterium
Subjects without
Fusobacterium
Total subjects
Number
236
120
116
Male/Female
177/59
98/22
79/37
Age
(years, mean±SD)
41.4 ± 11.9
(Range: 19–65)
40.6 ± 12.1
(Range: 19–64)
42.3 ± 11.6
(Range: 20–65)
Body mass index
(kg/m2, mean±SD)
23.2 ± 4.2
(Range: 15.9–42.9)
23.7 ± 4.3
(Range: 16.6–39.3)
22.7 ± 3.9
(Range: 15.9–42.9)
Diseases
Hypertension
27 (11%)
19 (16%)
8 (7%)
Dyslipidemia
44 (19%)
20 (17%)
24 (21%)
Diabetes
7 (3%)
6 (5%)
1 (0.9%)
Ulcerative colitis
0 (0%)
0 (0%)
0 (0%)
Crohn’s disease
0 (0%)
0 (0%)
0 (0%)
Irritable bowel syndrome
2 (0.8%)
0 (0%)
2 (1.7%)
Cancer
1 (0.4%)
1 (0.8%)
0 (0%)
This table presents data from our previous cross-sectional study (ref. 18)

## Slide 2
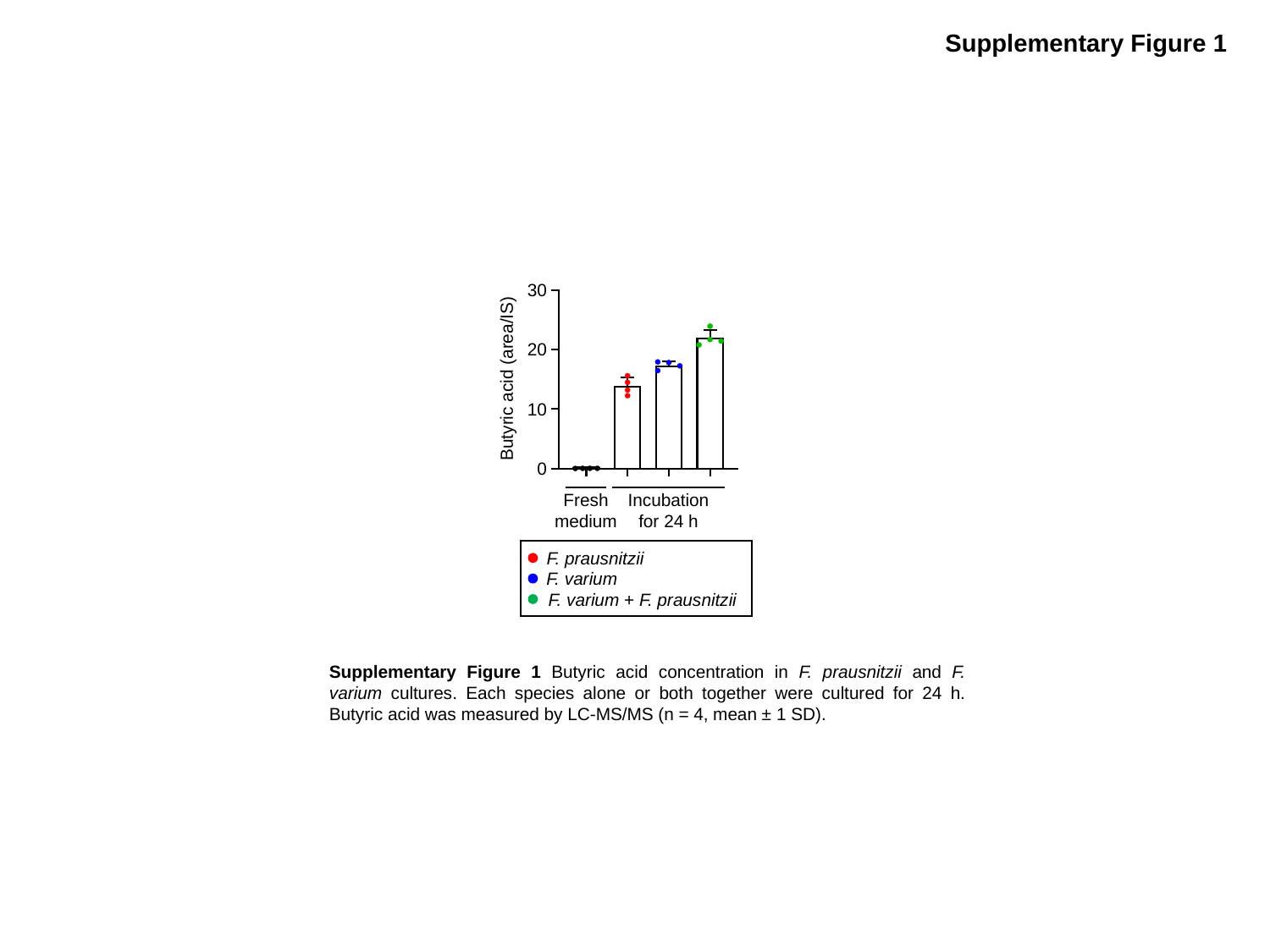

Supplementary Figure 1
30
20
Butyric acid (area/IS)
10
0
Fresh
medium
Incubation
for 24 h
F. prausnitzii
F. varium
F. varium + F. prausnitzii
Supplementary Figure 1 Butyric acid concentration in F. prausnitzii and F. varium cultures. Each species alone or both together were cultured for 24 h. Butyric acid was measured by LC-MS/MS (n = 4, mean ± 1 SD).

## Slide 3
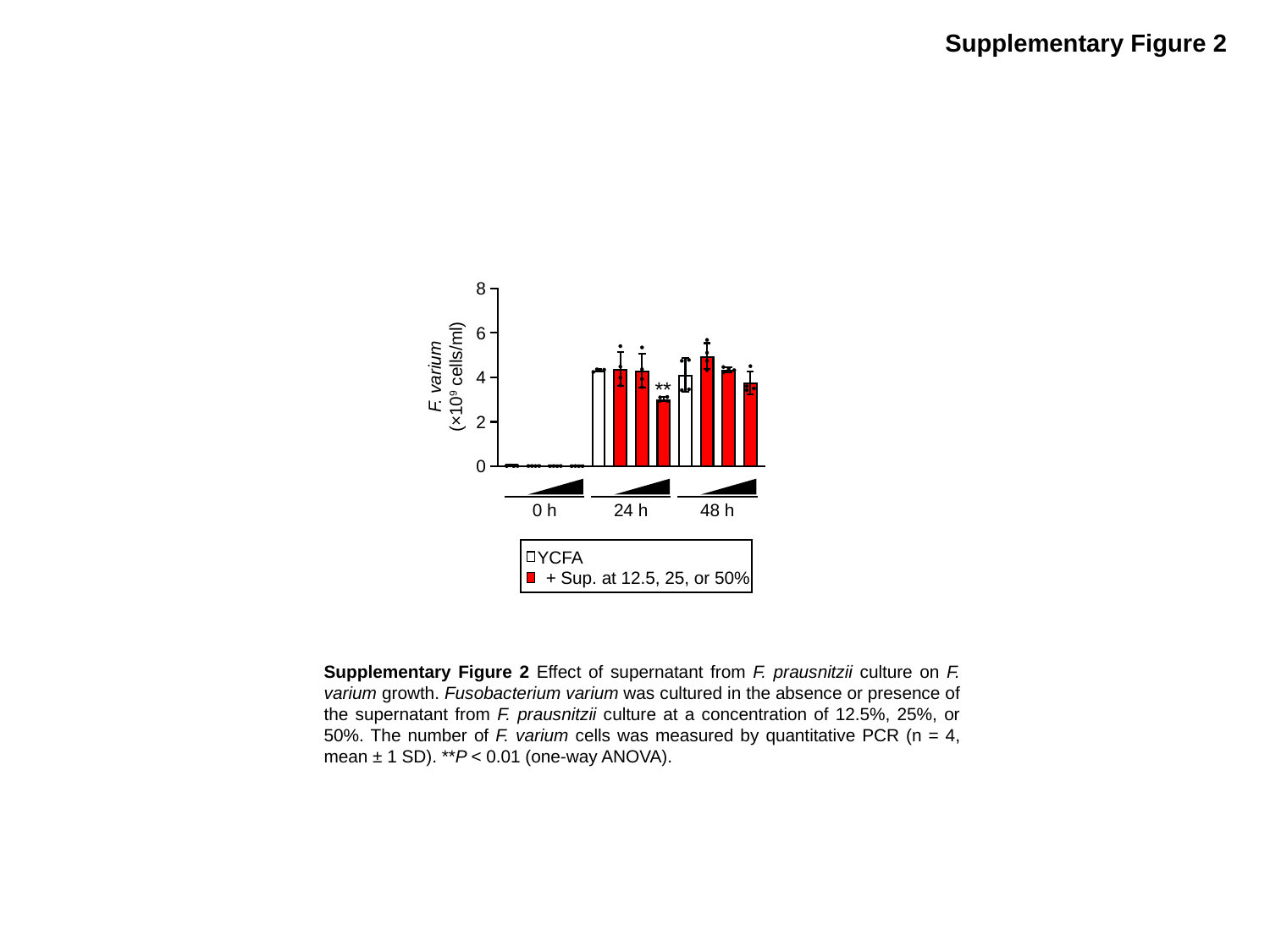

Supplementary Figure 2
8
6
F. varium
(×109 cells/ml)
4
2
0
**
0 h
24 h
48 h
YCFA
+ Sup. at 12.5, 25, or 50%
Supplementary Figure 2 Effect of supernatant from F. prausnitzii culture on F. varium growth. Fusobacterium varium was cultured in the absence or presence of the supernatant from F. prausnitzii culture at a concentration of 12.5%, 25%, or 50%. The number of F. varium cells was measured by quantitative PCR (n = 4, mean ± 1 SD). **P < 0.01 (one-way ANOVA).

## Slide 4
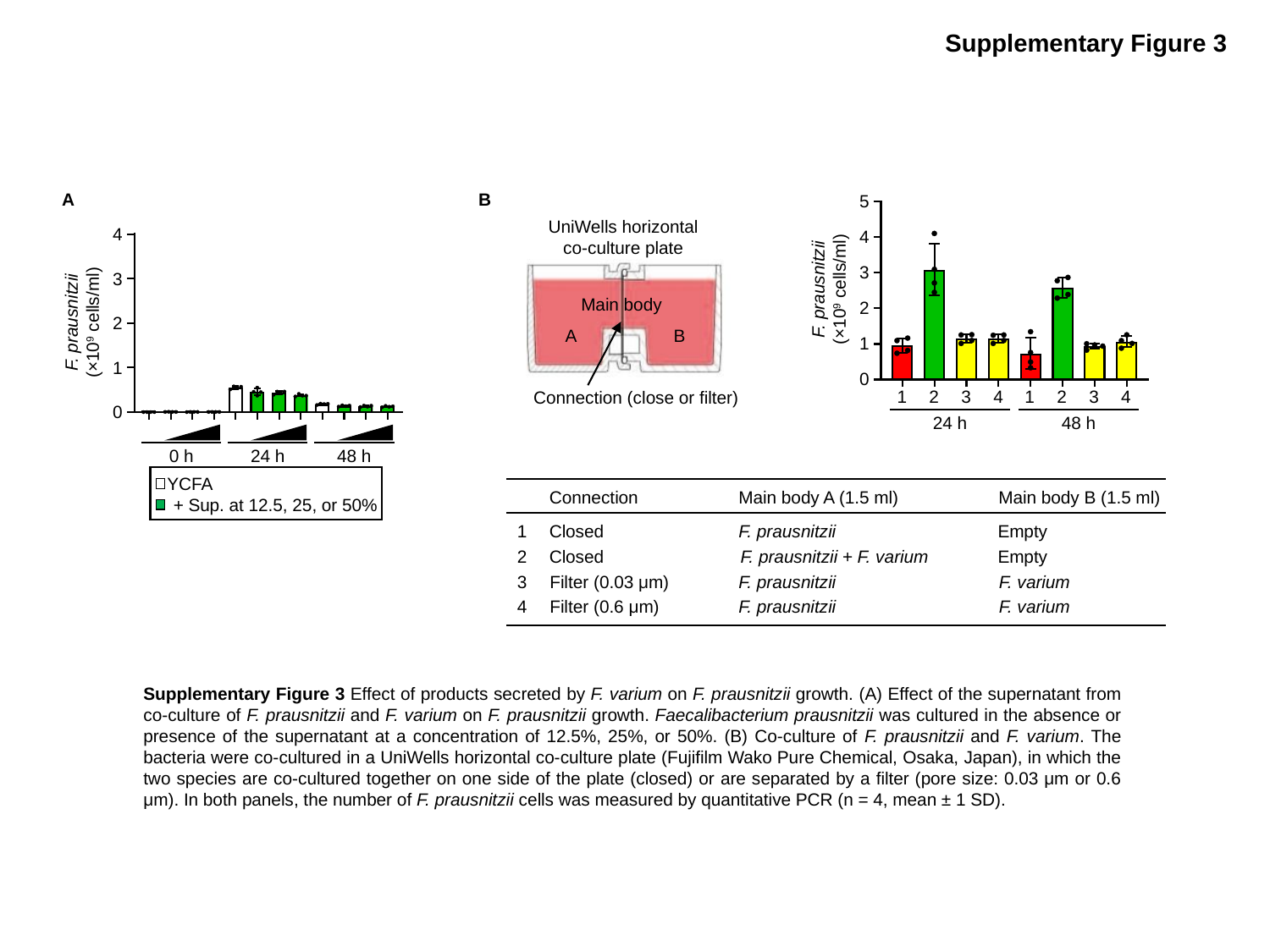

Supplementary Figure 3
A
B
5
4
3
F. prausnitzii
(×109 cells/ml)
2
1
0
0 h
24 h
48 h
YCFA
+ Sup. at 12.5, 25, or 50%
UniWells horizontal
co-culture plate
4
3
A
B
F. prausnitzii
(×109 cells/ml)
Main body
2
1
0
1
2
3
4
1
2
3
4
Connection (close or filter)
24 h
48 h
Connection
Main body A (1.5 ml)
Main body B (1.5 ml)
1
Closed
F. prausnitzii
Empty
2
Closed
F. prausnitzii + F. varium
Empty
3
Filter (0.03 μm)
F. prausnitzii
F. varium
4
Filter (0.6 μm)
F. prausnitzii
F. varium
Supplementary Figure 3 Effect of products secreted by F. varium on F. prausnitzii growth. (A) Effect of the supernatant from co-culture of F. prausnitzii and F. varium on F. prausnitzii growth. Faecalibacterium prausnitzii was cultured in the absence or presence of the supernatant at a concentration of 12.5%, 25%, or 50%. (B) Co-culture of F. prausnitzii and F. varium. The bacteria were co-cultured in a UniWells horizontal co-culture plate (Fujifilm Wako Pure Chemical, Osaka, Japan), in which the two species are co-cultured together on one side of the plate (closed) or are separated by a filter (pore size: 0.03 μm or 0.6 μm). In both panels, the number of F. prausnitzii cells was measured by quantitative PCR (n = 4, mean ± 1 SD).

## Slide 5
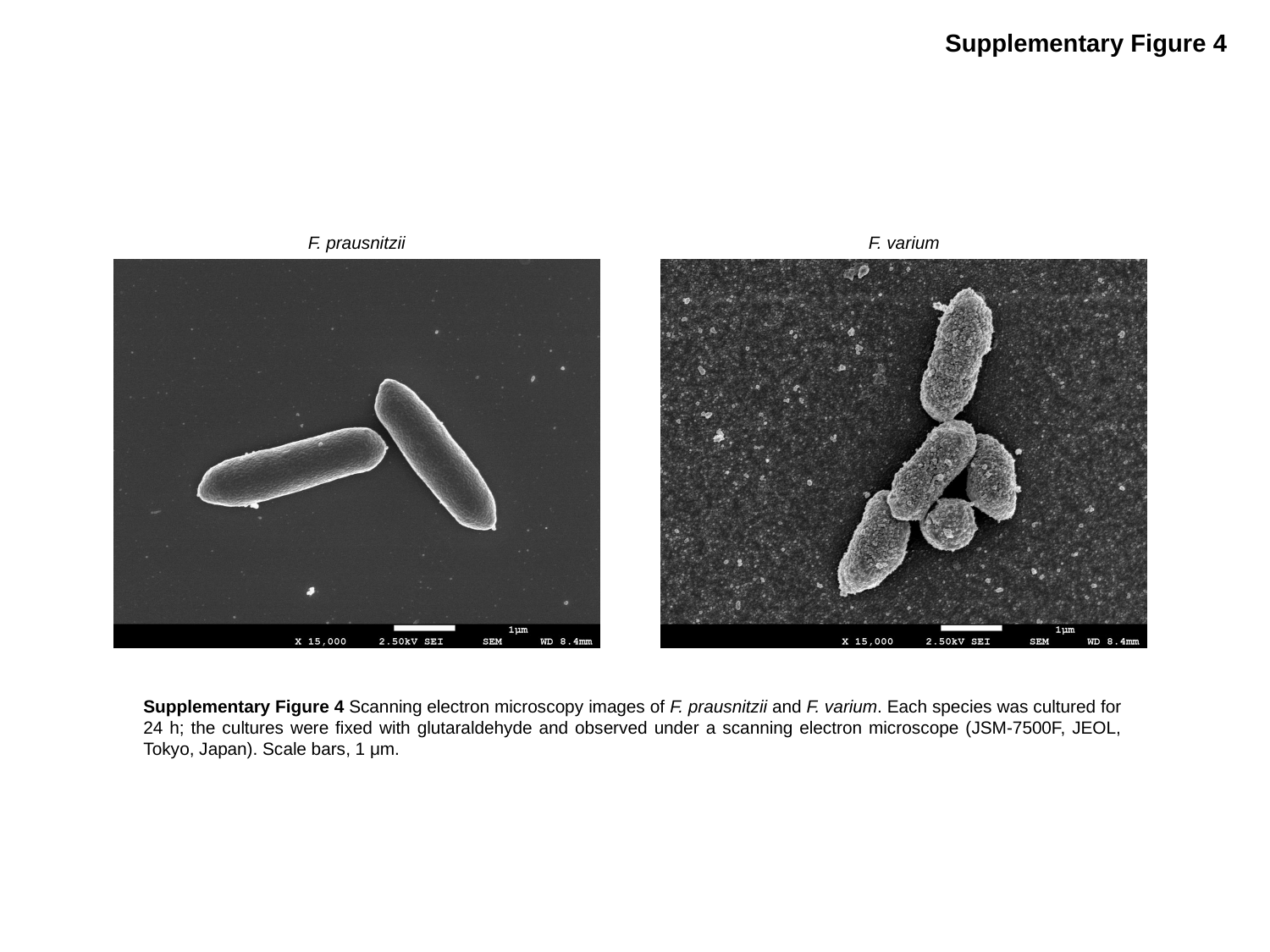

Supplementary Figure 4
F. prausnitzii
F. varium
Supplementary Figure 4 Scanning electron microscopy images of F. prausnitzii and F. varium. Each species was cultured for 24 h; the cultures were fixed with glutaraldehyde and observed under a scanning electron microscope (JSM-7500F, JEOL, Tokyo, Japan). Scale bars, 1 μm.
